# Glutamine supplementation in a child with inherited GS deficiency improves the clinical status and partially corrects the peripheral and central amino acid imbalance

**DOI:** 10.1186/1750-1172-7-48

**Published:** 2012-07-25

**Authors:** Johannes Häberle, Noora Shahbeck, Khalid Ibrahim, Bernhard Schmitt, Ianina Scheer, Ruth O’Gorman, Farrukh A Chaudhry, Tawfeg Ben-Omran

**Affiliations:** 1 Division of Metabolism and Children’s Research Center, University Children’s Hospital Zurich, 8032, Zurich, Switzerland; 2 Section of Clinical and Metabolic Genetics, Doha, Qatar; 3 Section of Pediatric Neurology, Department of Pediatrics, Hamad Medical Corporation, Doha, Qatar; 4 Division of Neuropediatrics, University Children’s Hospital Zurich, Zurich, Switzerland; 5 Division of Radiology, University Children’s Hospital Zurich, Zurich, Switzerland; 6 Center for Magnetic Resonance Research, University Children’s Hospital Zurich, Zurich, Switzerland; 7 The Biotechnology Centre and The Centre for Molecular Biology and Neuroscience, University of Oslo, Oslo, Norway; 8 Department of Pediatrics and Genetic Medicine, Weil-Cornell Medical College, New York, USA; 9 Department of Pediatrics and Genetic Medicine, Weil-Cornell Medical College, Doha, Qatar

**Keywords:** Glutamine supplementation, Glutamine synthetase, Chronic encephalopathy, Neonatal onset seizures, Hyperammonemia, Qatar consanguinity, Therapeutic trial, GABA, Neurotransmitter replenishment, SLC38

## Abstract

Glutamine synthetase (GS) is ubiquitously expressed in mammalian organisms and is a key enzyme in nitrogen metabolism. It is the only known enzyme capable of synthesising glutamine, an amino acid with many critical roles in the human organism. A defect in *GLUL*, encoding for GS, leads to congenital systemic glutamine deficiency and has been described in three patients with epileptic encephalopathy. There is no established treatment for this condition.

Here, we describe a therapeutic trial consisting of enteral and parenteral glutamine supplementation in a four year old patient with GS deficiency. The patient received increasing doses of glutamine up to 1020 mg/kg/day. The effect of this glutamine supplementation was monitored clinically, biochemically, and by studies of the electroencephalogram (EEG) as well as by brain magnetic resonance imaging and spectroscopy.

Treatment was well tolerated and clinical monitoring showed improved alertness. Concentrations of plasma glutamine normalized while levels in cerebrospinal fluid increased but remained below the lower reference range. The EEG showed clear improvement and spectroscopy revealed increasing concentrations of glutamine and glutamate in brain tissue. Concomitantly, there was no worsening of pre-existing chronic hyperammonemia.

In conclusion, supplementation of glutamine is a safe therapeutic option for inherited GS deficiency since it corrects the peripheral biochemical phenotype and partially also improves the central biochemical phenotype. There was some clinical improvement but the patient had a long standing severe encephalopathy. Earlier supplementation with glutamine might have prevented some of the neuronal damage.

## Introduction

Glutamine synthetase (GS) is a ubiquitously expressed cytosolic key enzyme of nitrogen metabolism [1,2]. In an ATP-dependent manner, GS catalyzes the synthesis of glutamine from glutamate and ammonia and is a source for the entire body glutamine pool, since there is no net absorption of glutamine from the intestine in healthy individuals [3,4]. The synthesis of glutamine from GS is the only known reaction for endogenous glutamine production in mammals, and GS also plays an important role in many metabolic pathways e.g. by balancing the nitrogen pool between different compartments, by detoxifying ammonia and glutamate in brain astroglial cells [5,6] or by providing osmotic cell homeostasis [6-8]. GS is expressed *in utero* in early human fetuses [9] and is a prerequisite for early development in mice and drosophila [10,11]. The product of the GS reaction, glutamine, is the major amino moiety donor for other amino acids, glucose precursors, purines and pyrimidines, and adenosine-monophosphate [12] and is the central metabolite for the temporary storage and transport of nitrogen between different organs [1]. Glutamine has a functional impact on many metabolic processes in peripheral organs, such as pH homeostasis in the kidney and regulation of plasma glucose levels in the pancreas [13,14]. In the central nervous system, glutamine is considered to be the primary precursor of the neurotransmitter amino acids glutamate and γ-aminobutyric acid (GABA), the most abundant fast excitatory and inhibitory neurotransmitters, respectively [15-17].

The pivotal roles of glutamine underline the importance of a functional GS for normal human physiology, so it is not surprising that patients with an inherited defect of GS demonstrate a severe clinical phenotype. These patients either died from neonatal multi-organ failure [18,19] or, as in the case of the patient described in this study, survived albeit suffering from severe epileptic encephalopathy [20]. Unfortunately, there is no established treatment for GS deficiency but supplementation of the missing amino acid would be a first-thought rationale. Glutamine supplementation has already been tested in healthy volunteers and doses up to 750 mg/kg/d have proved to be safe during short-term application [3,21-24]. However, since chronic hyperammonemia may be a consequence of GS deficiency [20], supplying glutamine to a patient with GS deficiency could augment ammonia levels, potentially causing additional toxicity [25]. Therefore, unlike therapy in the other amino acid biosynthesis disorders [26,27], glutamine supplementation must be carefully considered in the light of the potentially damaging effects of hyperammonemia. A careful therapeutic trial with thorough monitoring was necessary in order to establish the safety of glutamine supplementation.

In the current study, we present a clinical trial in the only known living GS deficient patient with severe neurological sequelae. We demonstrate that regulated glutamine supplementation with careful monitoring enables correction of the biochemical defect and provides some improvement in the neurological status of the patient.

## Methods

### Patient – clinical situation

The natural course of the patient has been described previously [20]. In brief, he was born to first cousin parents from Sudan after an uneventful pregnancy. At birth, he did not need resuscitation, but soon he was noted to have generalized hypotonia, lower limb hyperreflexia, cloni and episodes of eye staring lasting for 2–3 minutes. Generalized tonic-clonic convulsions started at 13 days and, despite extended anticonvulsant therapy, epileptic seizures persisted, contributing to a severe developmental delay. In addition, the patient demonstrated one episode of necrolytic erythema at 38 months [20] when serum glutamine levels were < 10 μmol/L.

At the start of the therapeutic trial, the patient was in a stuporous state with only a few episodes of apparent alertness during the day that each lasted about 15 minutes. If awake, the parents were able to identify emotional expressions such as satisfaction, hunger or anger. He was mainly fed via his gastrostomy tube but was also able to suck small amounts of his diet. Convulsions were noted every day, but it was unclear whether all should be classified as epileptic seizures. Overall, the patient had a robust constitution and a clinically intact immune system.

Before the start of the therapeutic trial, detailed clinical and nutritional assessments and biochemical investigations were performed at Hamad Medical Corporation, Doha-Qatar. In an attempt to lower plasma ammonia as much as possible before the initiation of the glutamine therapy, the patient received increasing doses of lactulose and neomycin to limit ammonia uptake from the gut. This was done under close monitoring for dehydration and electrolyte imbalances and resulted in a short-term decrease of plasma ammonia to 38 μmol/L. However, as diarrhoea became troublesome, this treatment was no longer tolerated and had to be stopped. The patient was then hospitalized for four weeks ensuring close clinical, laboratory and technical monitoring. The parents of the patient consented to the therapeutic trial which was performed at the University Children’s Hospital Zurich. The study was approved by the responsible institutional review board (Kantonale Ethikkommission).

### Biochemical investigations at baseline and during monitoring

Before glutamine supplementation was initiated, baseline metabolic and endocrinologic investigations were done in blood (including fasting plasma amino acid profile, ammonia, growth hormone, thyroid function, vitamins A, E, D and B12, and essential fatty acids) and in cerebrospinal fluid (CSF) (including amino acid profile, free and total GABA, neurotransmitter metabolites, folates, pterines, creatine, guanidinoacetate), in addition to routine biochemical parameters (such as blood count, serum electrolytes, creatinine, ASAT, and ALAT). Amino acid analysis in plasma and CSF was done applying the classical method based on ion exchange chromatography with post column derivatization with ninhydrin using the Biochrom 30+ analyzer (Biochrom, Cambridge, UK). Most metabolic parameters (including tests in CSF) were done weekly, but fasting plasma ammonia and amino acid profiles were obtained daily.

### Technical investigations at baseline and during monitoring

EEG examinations were recorded both in the awake and sleeping state at baseline and at weekly intervals during trial monitoring.

Cerebral magnetic resonance imaging (MRI) and spectroscopy (MRS) studies were performed with a 3 T GE HD.xt MRI scanner (GE Healthcare, Waukesha, WI, USA). The imaging protocol included one axial T1 weighted sequence (repetition time TR = 620 ms; echo time TE = 21 ms), T2 weighted sequences in 3 planes (TR = 9300 ms; TE = 96 ms), FLAIR (TR = 9300 ms; TE = 124 ms; inversion time TI = 2250 ms), diffusion weighted images DWI (35 gradient directions and *b_value* = 700 s/mm^2^, voxel = 0.8 x 0.8 x 2.5 mm^3^) and a T1 weighted sequence after intravenous Gadolinium injection.

Single voxel ^1^ H]MRS spectra were acquired from a voxel of interest in the left basal ganglia (15x15x15 mm^3^), using a point-resolved spectroscopy (PRESS) sequence with repetition time (TR) = 3000 ms and echo time (TE) = 35 ms. In order to examine the cerebral concentrations of GABA, glutamate, and glutamine in more detail, additional edited spectra were acquired from a 25x40x25 mm^3^ voxel of interest (VOI) in the left basal ganglia using a MEGA-PRESS sequence [28] with TR = 1800 ms, TE = 68 ms. For each metabolite spectrum, 16 water reference lines were also acquired as part of the standard PROBE acquisition. Metabolite concentrations were derived with LCModel version 6.1-4 F [29], using the unsuppressed water signal as an internal calibration standard. Following this method, the spectrum was modeled as a linear combination of basis spectra from in vitro metabolite solutions of known concentration, (see reference [29] for further details). Since this method incorporates the full multiplet peak profiles for glutamate and glutamine, the relative contributions from these neurotransmitters can be resolved despite their overlapping peaks. Brain MRI and MRS studies were repeated after 2 and 4 weeks, respectively. For comparison, the same MRS protocol was applied in three age-matched comparison subjects, referred for MRI for skull malformation, developmental delay, and epilepsy, respectively, with normal cerebral MR imaging.

### Glutamine supplementation

**Figure 1:**
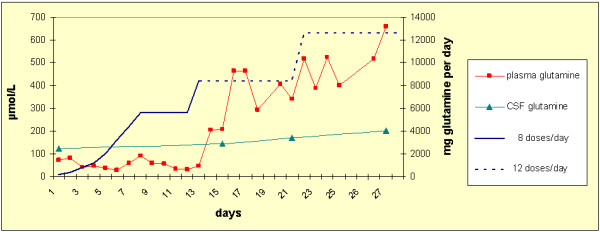
**Glutamine dosing regimen and plasma concentrations during the 4 weeks trial.** Glutamine was initially given 8 doses per day (blue line) but switched to 12 doses per day (blue dotted line) after 14 days treatment. Fasting plasma glutamine concentrations are given (with red ▪) as obtained every morning and for CSF glutamine concentrations (with turquoise ) illustrating the increase in plasma and CSF glutamine concentrations, respectively, under glutamine supplementation.

L-glutamine was provided as a powder (Resource® Glutamin, Nestlé Nutrition, Vevey, Switzerland). A low starting regime of 17 mg/kg/d divided in eight dosages was chosen because of concerns about the possible toxicity of raising ammonia and glutamate levels. Glutamine was then increased step-wise each day to a final dose of 1020 mg/kg/on day 21 of the trial. Since fasting glutamine levels did not increase during the first two weeks of the trial, the interval between single dosages was shortened to two hours (Figure 1). To facilitate the treatment, 330 mg/kg glutamine was administered from 12 p.m. until 8 a.m. as a continuous enteral infusion via the gastrostomy tube after dilution in 100 ml water. Prior to this infusion, the stability of the glutamine solution was confirmed by measurement of glutamine at 0, 6 and 9 hours, which yielded steady concentrations (data not shown). To provide information on the parenteral dose of glutamine needed for maintaining normalized plasma glutamine levels, L-glutamine (Dipeptiven®, Fresenius Kabi, Stans, Switzerland) was given as a continuous infusion over six hours at a rate of 21 and 31 mg/kg/h, respectively, and over eight hours at a rate of 41 mg/kg/h while enteral glutamine supplementation was paused.

## Results

### Clinical situation

The therapeutic trial lasted for four weeks. On one occasion the patient suffered from a catheter-associated infection requiring targeted intravenous antibiotics (amoxicillin clavulanate, gentamicin). However, the patient remained clinically stable throughout this four week period.

Two weeks after the start of glutamine supplementation, the plasma glutamine concentrations increased with a concomitant improvement in the patient’s alertness. This increased alertness was noted by the parents but also by staff members based on prolonged awake phases. In addition, the intensity of smiling of the patient improved resulting in his “longest ever smile” on the last day of the trial as described by the father.

Both enteral and parenteral L-glutamine supplementation was tolerated during the four week trial without side effects. However, when the patient returned to his home country, the parents noticed an increased irritability. Since this was attributed to L-glutamine supplementation, the dose of glutamine was reduced again, however, without accurate documentation.

### Monitoring of biochemical parameters

Considerable concern was focused on the levels of ammonia and glutamate, which remained stable in blood as well as in CSF (Table 1). Fasting concentrations of plasma glutamine were 74 μmol/L (reference range 457–857) at the start of the trial, remaining stable while glutamine was given every three hours, but increasing above the lower limit of normal when glutamine was given at a dose of 700 mg/kg/d at a two hourly interval (Figure 1). There was no clear decrease of plasma glutamine related to the catheter-associated infection, but this might have escaped our only once-daily monitoring. Levels of CSF glutamine constantly increased from 121 at baseline to 146, 171 and 201 μmol/L (reference range 333–658) after 2, 3, and 4 weeks of treatment, respectively, but remained below the lower limit of normal in this limited trial period (Table 1 and Figure 1).

**Table 1:** Relevant biochemical and MRS investigations at baseline and after four weeks glutamine supplementation

**Parameter**	**Baseline**	**End of trial**	**Reference range**
***Blood***			
Ammonia (μmol/L)	**66**	**76**	12-48
Glutamine (μmol/L)	**74**	731	457–857
Glutamate (μmol/L)	58	28	17-69
***CSF***			
Ammonia (μmol/L)	**71**	**75**	12-48
Glutamine (μmol/L)	**121**	**201**	333–658
Glutamate (μmol/L)	<2.5	<2.5	0–5
Homovanillic acid (nmol/L)	**171**	**188**	211–871
Creatine (μmol/L)	84	56	17–87
Free GABA (μmol/L)	0.030	0.037	0.032-0.167
Total GABA (μmol/L)	8.3	7.47	3.3-12.2
***Basal ganglia MRS***			*Reference values from age-matched comparison subjects (n = 3)*
NAA (IU)	**4.2**	**4.6**	8.3-8.8
Creatine + phosphocreatine (IU)	**12.0**	**10.5**	8.4-8.6
Choline (IU)	**3.6**	**2.9**	1.7-2.0
Myo-inositol (IU)	**6.7**	**6.4**	3.0-4.0
Glutamate (IU)	**4.6**	**4.9**	7.3-10.3
Glutamine (IU)	**0**	**1.4**	3.1-6.3
GABA (IU)	3.0	2.6	2.4-3.7

Pathological values in bold; GABA: γ-aminobutyric acid; NAA: N-acetylaspartate.

Concentrations of creatine and guanidinoacetate in CSF were normal in this patient both at baseline and at the end of the trial. However, baseline creatine was in the upper normal range and showed a similar decrease to that observed by MRS. CSF concentrations of homovanillic acid (171 and 188 nmol/L, respectively, reference range 211–871) were unchanged during the trial. Levels of CSF folates and pterines were normal (data not shown) as were concentrations of glutamate and total as well as free GABA both before and at the end of the trial (Table 1).

### Monitoring of EEG, MRI and MRS

**Figure 2:**
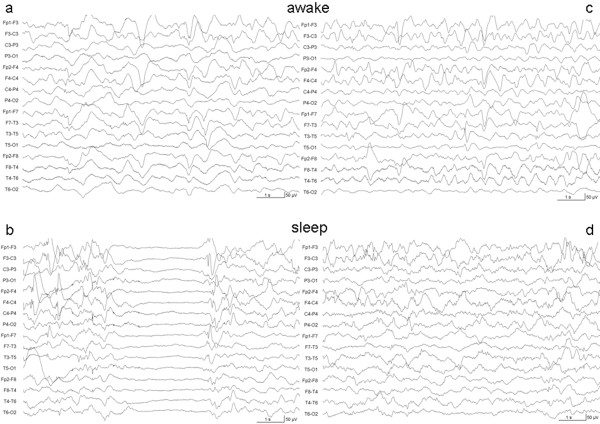
**EEG before and at the end of the therapeutic trial.** EEG recordings before supplementation of glutamine (2**a,b**) and at the end of the therapeutic trial (2**c,d**) showing the significant improvement of the background activity and the reduction of multifocal sharp waves both in the awake state (2**c**) and during sleep (2**d**).

EEGs recorded at baseline were severely abnormal with frontal high amplitude slow waves (0.5-2/s) and intermittent generalized subdelta (0.5/s) waves. During sleep, amplitude was intermittently attenuated in irregular intervals and partly suppressed for 1–3 seconds. Multifocal sharp waves were present when awake and more frequently when asleep. EEG after 1 week of treatment showed a slightly improved background activity and the intermittent amplitude attenuation during sleep seemed to be less distinct. After 3 weeks of glutamine treatment the background activity had clearly improved with dominant 3-5/s activity in the awake state. After 4 weeks, intermittent amplitude attenuations completely disappeared during sleep and multifocal sharp waves were less frequent both awake and asleep (Figure 2).

**Figure 3:**
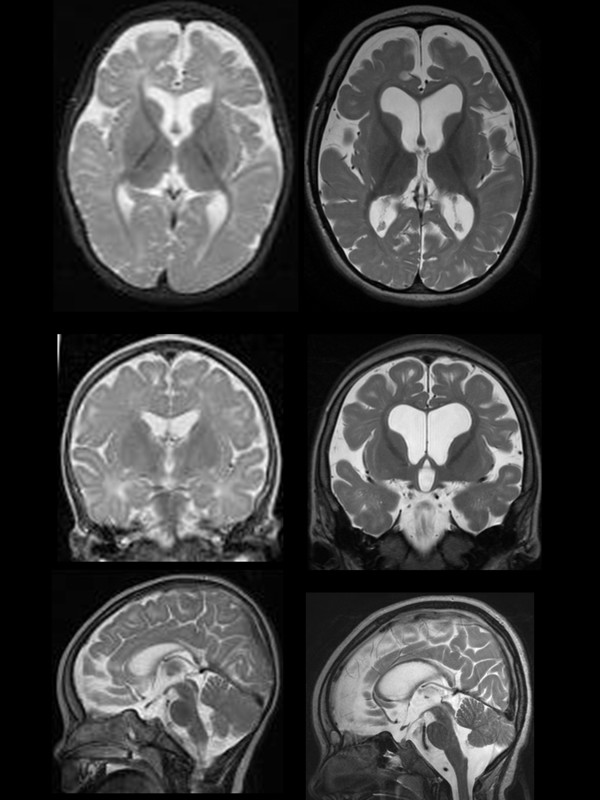
**MRI investigations.** T2 weighted images obtained at age 11 months (left column) and at the beginning of the therapeutic trial (at age 3 ^9/12^ years; right column) illustrating the severe brain atrophy caused by loss of cerebral white matter with consequent thinning of the corpus callosum.

The baseline MRI (performed at age 3 ^9/12^ years) showed changes that were not present in the previous examination at 11 months [20], including increased white matter signal intensity on T2 weighted images, prominent Virchow-Robin-spaces, reduced white matter volume with consequent thinning of the corpus callosum and secondary enlargement of CSF spaces (see Figure 3). The basal ganglia were atrophic and no contrast enhancement was present. The two following MRI exams did not show further changes (data not shown). 

**Figure 4:**
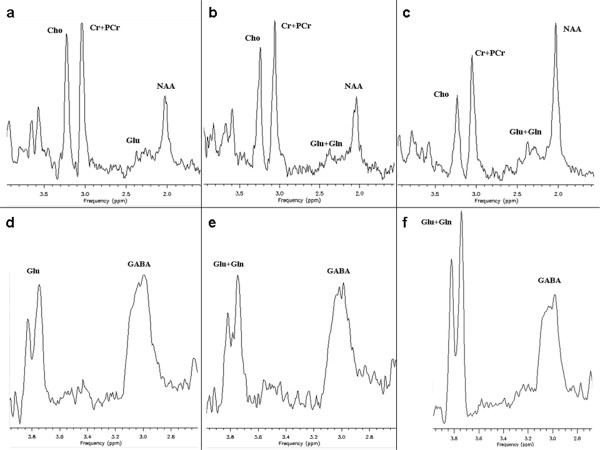
**MRS investigations.** Brain MRS obtained from the patient at baseline (4a, d) and at the end of the therapeutic trial (4b,e). In addition, an average spectrum from 3 comparison subjects is shown (4c, f). Note the absence of Gln (glutamine) at baseline (4a, d) and the increase of Glu + Gln (glutamate and glutamine) at the end of the trial (4b,e). Figures 4a-c show the spectra acquired with the standard PRESS sequence, and figures 4d-f show the MEGA-PRESS data.

At baseline, MRS demonstrated undetectable glutamine, reduced N-acetylaspartate (NAA) and glutamate and elevated creatine, choline, and myo-inositol (mI). Monitoring after two weeks revealed increased glutamine and glutamate levels and a decrease of creatine and choline with a modest increase in NAA. After four weeks, glutamine, glutamate, and NAA levels continued to increase but remained below normal. Creatine and choline levels continued to decrease but remained above normal. GABA levels decreased during the trial but remained within the normal range (Figure 4).

## Discussion

### Glutamine supplementation is well tolerated and partially rescues the biochemical phenotype

With this study, we provide the first report of a therapeutic trial for a patient with congenital GS deficiency. The patient is the only known living patient with this disorder [20], as the two other patients previously reported [18,19] did not survive beyond the neonatal age, rendering any therapy impossible. A plausible but not proven explanation for the survival of this patient would be the assumption of a slightly higher level of GS residual activity due to another missense mutation of the *GLUL* gene (p.Arg324Ser in the case of the present patient [20]; p.Arg324Cys and p.Arg341Cys in the case of the neonatally deceased patients [18]). Supplementation of an amino acid has already been successfully applied in other amino acid biosynthesis disorders, both in a prophylactic approach during pregnancy and in a therapeutic way postnatally [26,27,30]. Thus, supplementation of L-glutamine is a logical therapeutic approach, but was burdened with concerns about possible increases in ammonia and extracellular glutamate concentrations, potentially leading to neurotoxicity and increased susceptibility to seizures [31].

As the main result of this trial, the intervention was well tolerated and allowed the normalization of the peripheral biochemical phenotype of GS deficiency, as demonstrated by normal plasma glutamine levels under high-dose glutamine supplementation (1020 mg/kg/d). This observation confirms earlier reports that while at normal levels of intake there is no net absorption of glutamine in the intestine of healthy individuals, additional supplementation does increase plasma glutamine concentrations [3,21,32]. However, in GS deficiency there is presumably some net absorption at lower levels of glutamine intake. In addition, the central biochemical phenotype could be substantially improved, as demonstrated by enhanced glutamine availability in CSF and brain tissue. Brain MRS based quantification of glutamine yielded concentrations that continuously increased from zero to measurable levels, albeit still below the findings seen in age-matched comparison subjects. MRS glutamate was also low at baseline, increasing during the trial but still remaining low relative to typical levels. Since MRS measurements of glutamate, glutamine and GABA do not provide information concerning their subcellular localisation, it is not known if the increases in glutamine and glutamate detected with MRS reflect changes in the neurotransmitter pools of these metabolites or to changes in metabolic pools. However, the lack of a further accumulation of glutamine in brain tissue is consistent with an increase in utilisation. Lack of total restoration of the biochemical phenotype in the CNS may partially be due to brain atrophy as shown in the basal ganglia by MRI. Atrophy and/or neurodegeneration are also supported by the low levels of NAA and the elevated levels of creatine and choline demonstrated by MRS and CSF measurements.

In normal subjects, the glutamine transporter SN1 (SLC38A3) at the astroglial membranes maintains glutamine concentrations in the extracellular fluid around 0.4 mM by bidirectional glutamine transport [33,34]. With a dysfunctional GS the astroglial cells no longer produce glutamine and their contribution to the extracellular glutamine homeostasis is abolished. With glutamine supplementation, glutamine will cross the blood–brain barrier and increase the levels of glutamine in the perisynaptic extracellular compartments [35]. Many of the neuronal glutamine transporters - including the unidirectional system A transporters SAT1 (SLC38A1) and SAT2 (SLC38A2) shown to be fundamental for neurotransmitter generation [17,36,37] - have Km values in the order of 0.2-2.3 mM for glutamine [38,39]. These transporters will be stimulated by the increased supply of glutamine, enabling the neurons to accumulate glutamine continuously and facilitating the catabolism of glutamine to sustain synthesis of the neurotransmitters glutamate and GABA [36]. Thus, lack of further build-up of glutamine in brain tissue and in CSF is consistent with its increased utilization. Furthermore, our data suggest that glutamine supplementation counteracts the imbalance in glutamatergic and GABAergic drive described in some forms of epilepsy [40] and we speculate that this might form the basis of the improvement in the background EEG activity, reflecting the clinical amelioration of the encephalopathy. Thus, our data show that glutamine supplementation succeeded in increasing the supply of glutamine throughout the body, possibly stimulating cellular metabolism and sustaining normal function.

### Glutamine supplementation does not aggravate hyperammonemia

As demonstrated in this study, levels of plasma and CSF ammonia remained stable even when glutamine supplementation was increased to the maximum of 1020 mg/kg/d. The slightly higher plasma ammonia concentrations at the end of the trial are likely attributed to the infection, as concomitant CSF ammonia levels remained unchanged in comparison to baseline levels (Table 1). Our results correspond well to data gained from genetic disruption of GS in mice which show a modest increase (1.6 fold) in cortical ammonia levels [41]. However, in addition, we show that a therapeutic intervention with glutamine supplementation does not exacerbate the levels of plasma and CSF ammonia in a human. Unfortunately, there was no close monitoring of ammonia (or amino acid profiles) after the discharge of the patient from the hospital when he developed irritability, so no conclusions can be drawn with regard to the cause of this irritability.

### Glutamine is critically needed during pregnancy, in the neonatal and early childhood periods

The severe changes in cerebral white matter at the beginning of the therapeutic trial (Figure 3) demonstrate that the brain of the patient has probably deteriorated over the years due to glutamine deficiency and lack of its metabolic products, and due to severe epileptic encephalopathy. This observed neurodegeneration is consistent with experimental findings supporting the role of GS for Schwann cell differentiation [42]. During pregnancy, there is a great need for glutamine for normal ontogeny and brain development [43,44] and there is GS-dependent de novo synthesis of glutamine in the placenta [45]. As the glutamine transporters on the placenta are also upregulated to transport glutamine actively from the maternal circulation to the fetus the demand may be partially met by increased extraction of glutamine from the maternal circulation [46]. However, this supply is abolished after birth, while the demand for glutamine – e.g. for glutamate and GABA formation and myelination - continues to increase due to synaptogenesis and establishment of functional neuronal networks [47].

Substantial clinical improvement in the patient’s condition was therefore unlikely. However, the improvement in alertness of the patient, although difficult to quantify for external evaluation, can be considered as encouraging with respect to an earlier start of treatment in future patients. Likewise, EEG recordings were much improved both in the awake and sleeping state, suggesting that external glutamine can cross the blood brain barrier and possibly influence neuronal activity. Of note, enteral glutamine supplementation was sufficient to establish and maintain normal plasma glutamine levels. As an alternative if enteral use is not feasible, parenteral application was also possible in this patient but was considered as a second choice given the higher invasiveness.

## Conclusions

In summary, this is the first report of a therapeutic intervention in a patient with an inherited defect of GS, demonstrating the feasibility of correcting systemic glutamine deficiency in this disorder without worsening pre-existing hyperammonemia or provoking toxic CSF glutamate levels. For future patients, glutamine supplementation should begin as early as possible in order to elucidate to what extent earlier intervention can prevent the devastating natural course of chronic encephalopathy in GS deficiency. In fact, since glutamine is a functionally essential amino acid also in healthy persons, adequate maternal production and supply of glutamine to the fetal circulation may be a limiting factor in particular in the presence of GS deficiency in the placenta. Thus, glutamine supplementation may have beneficial outcomes during pregnancy for a child with GS deficiency and should be considered. Since newborn screening programs will probably not adopt GS deficiency as a target disease based on its extremely low incidence and on the instability of glutamine in dried blood samples, a high awareness towards low glutamine levels found in selective screening is needed. Moreover, additional research is warranted to make headway towards ameliorating the effects of this devastating condition as well as to improve outcomes for this rare genetic disease.

## Competing interests

The authors declare that they have no competing interests.

## Authors’ contributions

All authors of this work have contributed to the planning and performing of the study as well as to writing and revising of this paper. All authors read and approved the final manuscript.
